# An Exploration of Common Greenhouse Gas Emissions by the Cyanobiont of the *Azolla–Nostoc* Symbiosis and Clues as to Nod Factors in Cyanobacteria

**DOI:** 10.3390/plants8120587

**Published:** 2019-12-10

**Authors:** Dilantha Gunawardana

**Affiliations:** Department of Botany, University of Sri Jayewardenepura, Gangodawila 10250, Sri Lanka; dilantha@sci.sjp.ac.lk

**Keywords:** *Azolla* spp., Nitric oxide reductases, Calvin Cycle, *Nostoc azollae*, Nod factors

## Abstract

*Azolla* is a genus of aquatic ferns that engages in a unique symbiosis with a cyanobiont that is resistant to cultivation. *Azolla* spp. are earmarked as a possible candidate to mitigate greenhouse gases, in particular, carbon dioxide. That opinion is underlined here in this paper to show the broader impact of *Azolla* spp. on greenhouse gas mitigation by revealing the enzyme catalogue in the *Nostoc* cyanobiont to be a poor contributor to climate change. First, regarding carbon assimilation, it was inferred that the carboxylation activity of the Rubisco enzyme of *Azolla* plants is able to quench carbon dioxide on par with other C_3_ plants and fellow aquatic free-floating macrophytes, with the cyanobiont contributing on average ~18% of the carboxylation load. Additionally, the author demonstrates here, using bioinformatics and past literature, that the *Nostoc* cyanobiont of *Azolla* does not contain nitric oxide reductase, a key enzyme that emanates nitrous oxide. In fact, all *Nostoc* species, both symbiotic and nonsymbiotic, are deficient in nitric oxide reductases. Furthermore, the *Azolla* cyanobiont is negative for methanogenic enzymes that use coenzyme conjugates to emit methane. With the absence of nitrous oxide and methane release, and the potential ability to convert ambient nitrous oxide into nitrogen gas, it is safe to say that the *Azolla* cyanobiont has a myriad of features that are poor contributors to climate change, which on top of carbon dioxide quenching by the Calvin cycle in *Azolla* plants, makes it an efficient holistic candidate to be developed as a force for climate change mitigation, especially in irrigated urea-fed rice fields. The author also shows that *Nostoc* cyanobionts are theoretically capable of Nod factor synthesis, similar to *Rhizobia* and some *Frankia* species, which is a new horizon to explore in the future.

## 1. Introduction

Three gases—carbon dioxide, methane (~34 times greater global warming potential than carbon dioxide), and nitrous oxide (a ~298-fold higher global warming potential than carbon dioxide)—are the chief contributors to global warming and, consequently, climate change [1,2]. The cultivation of crops or agriculture is one of the main causes of climate change, because it leads to the emission of all three of these gases. Heavy machinery and the Haber process, which is used to produce ammonia fertilizers, have high carbon footprints. Farming of ruminants and rice cultivation, which promote anaerobic decomposition, are both emitters of methane. Finally, the use of synthetic nitrogen fertilizers potentiates the denitrification of nitrogen compounds, which leads to the emission of nitrous oxide as a by-product. Therefore, it is necessary to reduce the effect of agriculture on climate change by promoting the use of biological alternatives to nitrogen fertilizers (biofertilizers), i.e., bacterial/cyanobacterial organisms that possess the remarkable ability to fix nitrogen gas and to furnish the nitrogen requirements of plants [3]. Nitrogen fixation is biologically viable, does not pollute or emit greenhouse gases, and is cost effective and user friendly, which makes this biological process a potent player in mitigating future climate change. Notably, among such alternative solutions is a plant, the aquatic fern *Azolla* sp., that can act as a biofertilizer by the fortification of a plant biological system with a nitrogen-fixing cyanobiont that churns out ammonia in abundance [3].

*Azolla* spp. can quench ample carbon dioxide due to their rapid proliferation, doubling their body weight in 2–5 days [4]. *Azolla* spp. are grown in paddy fields, where it has been measured that the yields of rice are significantly elevated due to dual cropping [5,6], while the lowering of the urea footprint is also a positive outcome based on the growth of *Azolla* spp. in irrigated lands. Urea has been shown to drastically improve nitrous oxide production while being a lesser candidate for the emission of methane; however, it increases the carbon dioxide footprint, which means that using biofertilizers as surrogates to urea diminishes climate change in three combined mechanisms [3,7]. At an ambient CO_2_ concentration of 800 ppm, *Azolla* spp., in a small-scale production system, showed a strong increase in biomass production, i.e., 36–47%, which establishes the potential of *Azolla* spp. as a solution to elevated atmospheric carbon dioxide levels [8]. *Azolla* spp. are C_3_ plants, and based on the first assimilation step by the action of the Rubisco carboxylase, they provide a heightened form of assimilation which would be negligible in C_4_ plants, where the first step is a concentrating/enrichment mechanism [9,10,11]. 

The production of copious amounts of ammonia via nitrogen fixation, which is then transported as amino acids such as glutamine, gives aquatic ferns abundant amounts of the Rubisco enzyme, which is the workhorse of plants carboxylating ribulose-1,5-disphosphate into carbon skeletons made of six carbon atoms [12]. In plants, Rubisco is said to compose ~30% of the total protein content, which may be even higher for an ammonium-churning engine such as the *Azolla* superorganism. Although rightfully considered a carbon quencher, this benefit would be made irrelevant by the production of more potent greenhouse gases such as nitrous oxide, methane, and halocarbons from the microbiome associated with the aquatic *Azolla* fern, mainly, the commonly-found cyanobiont which is popularly known as *Nostoc azollae*. It is therefore imperative to investigate the potential of both (1) the *Azolla* sp. (the host organism) and (2) the *Nostoc* major cyanobiont as contributors to greenhouse gas reductions. The major cyanobiont is known by three generic names, *Nostoc, Anabaena*, and *Trichormus* [13], but for the sake of simplicity and consistency, I have employed the name *N. azollae* throughout the article. 

This study focused mostly on the cyanobiont *N. azollae* and its paucity of de novo production of greenhouse gases, with the exception of carbon dioxide, which can be soaked/negated conceptually by the *Azolla* aquatic fern. I also shed light on the nature of putative signaling between the cyanobiont and host plant system using bioinformatics. To my knowledge, this is the first time that cyanobacteria have been shown (at the genome level) to possess putative Nod-factor-modifying enzyme encoding genes. 

This study only showcased the absence of key proteins for greenhouse gas production in the cyanobiont harbored inside *Azolla* spp. by using simple bioinformatics tools. The results suggest that *Azolla* spp. can be particularly useful for climate change mitigation, and this potential should be harnessed. This study will hopefully trigger physiological and climate change mitigation studies on the carbon fixation potential of *Azolla* species. 

## 2. Results and Discussion

### 2.1. Carbon Dioxide Emissions by the Cyanobiont N. azollae

**Figure 1:**
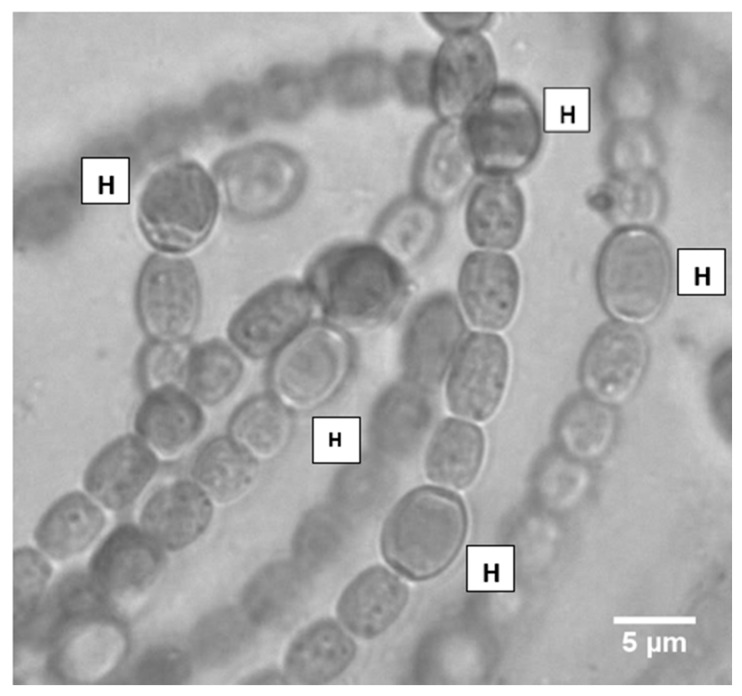
Heterocysts (H) of *Nostoc azollae*, where they are shown to have subtle differences in size, shape, and the smoothness/roughness of contours, and are spaced as close as four cells apart. Heterocysts are a major site of cellular respiration.

Due to the widespread presence of *N. azollae* inside *Azolla* fronds, it is unclear how much of the carbon dioxide that is emanated by *Nostoc* colonies is quenched by *Azolla* plants. It is known that in heterocysts, where nitrogen fixation occurs, there are strong respiration rates to maintain a low stream of oxygen at the active site of the nitrogenase enzyme, which means that heightened carbon dioxide emissions are a common occurrence from *Nostoc* filaments, especially in the dark. In this study on *N. azollae*, heterocysts were found as close as two cells apart, and were commonly spaced every 2–10 cells (Figure 1). The presence of a high number of heterocysts, compared with other *Nostoc* species (Table 1), means that *N. azollae* could be a potent contributor to greenhouse gas emissions and, consequently, climate change, unless the emanated carbon dioxide is quenched by photosynthesis (Calvin cycle) taking place in the *Azolla* fronds. 

Due to the high production of ammonium by nitrogen fixation in the filaments of *N. azollae*, *Azolla* plants, which benefit from the copious amounts of fixed nitrogen, can grow more rapidly, and quickly double their biomass. Ironically, the compartment (heterocyst) that synthesizes the nitrogen for Rubisco also produces high amounts of heterocystous carbon dioxide, which means that both the enzyme Rubisco and the substrate carbon dioxide emanate as physiological/biochemical products from the cyanobiont. 

*Azolla* cyanobionts have 5-fold less Rubisco, as quantified by their transcript numbers, compared with their free-living counterparts, and cyanobionts only have a partial/depleted carbon dioxide fixing ability [14]. This puts the responsibility of counteracting the carbon dioxide emanated by heterocysts—and overall, by *Nostoc* filaments—on the shoulders of the plant-type Rubisco enzyme. 

Rubisco is known to be present at around 30% of a plant’s catalogue of proteins. Here, however, I was unable to quantify the level of Rubisco in *Azolla* plants, which has not been indicated in the literature. It has been shown that carbon dioxide assimilation takes place at 90–100 µmol CO_2_/mg Chl.h for *Azolla* symbiosis and 75–80 µmol CO_2_/mg Chl.h for *Azolla* lacking the cyanobiont [15], which shows that photosynthesis is affected when the symbiosis is absent. Therefore, for maximum carbon assimilation, the cyanobiont is a critical component of the *Azolla* system. 

Furthermore, in C_3_ plants, there is 15–35 mg CO_2_/(dm^2^·h), while in C_4_ plants, there is 40–80 mg CO_2_/(dm^2^·h), which hints that the rates of carbon capture are relatively conservative in the *Azolla–Nostoc* system, even without quantifying the cyanobiont’s contribution [16]. On average, there is 3–5 mg of chlorophyll in 1 dm^2^ of leaf tissue, which is between 12–22 mg CO_2_/(dm^2^·h) for *Azolla*–cyanobiont, a conservative measurement for the plant system, being low to moderate among C_3_ counterparts, but similar to other floating aquatic plants that assimilate CO_2_ over the water surface at 20–23 mg CO_2_/(dm^2^·h) [17]. Still, this value may be higher, considering that the CO_2_ in the atmosphere is beyond 415 ppm now, and the aquatic fern’s carboxylation potential was measured in a study 40 years ago.

### 2.2. Oxides of Nitrogen

In this bioinformatics exploration, I examined the potential of the cyanobiont of *Azolla* spp. to be a nitrous oxide, methane, and halocarbon producer, compared to other members of the order Nostocales. I hypothesized that greenhouse-gas-producing enzymes were made redundant at the genome level in the *Azolla* cyanobiont, as it lost its independence and was put under the protection of the carbon nutrition of the host organism, which is a case of “genomic erosion” [20].

**Figure 2:**
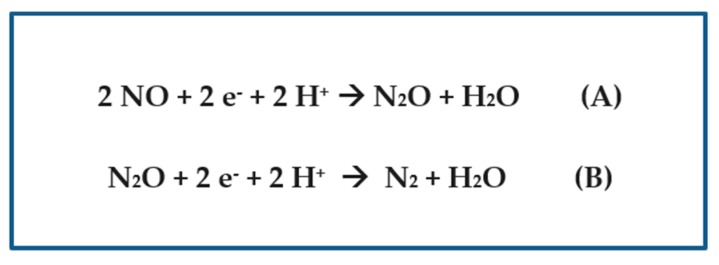
(**A**) The equation for the enzymatic conversion of nitric oxide into nitrous oxide. (**B**) The equation for the enzymatic conversion of nitrous oxide into elemental dinitrogen gas.

Regarding nitrous oxide production, I focused first on the nitric oxide reductase (GenBank: AFZ55274.1, belonging to *Cyanobacterium aponinum* PCC 10605) (Table 2) and the nitrous oxide reductase (RCJ21339.1, belonging to *Nostoc* sp. ATCC 43529) (Table 2), which catalyze sequential steps in the mechanism of denitrification, by using the PSI-BLAST search tool to unveil their presence (or absence) in their respective proteomes [21]. The reactions that these enzymes catalyze are shown in Figure 2. Surprisingly, the genus *Nostoc* (as well as the sibling *Anabaena*) was found not to possess any nitric oxide reductases that catalyze the production of the greenhouse gas nitrous oxide, but it does contain putative nitrous oxide reductases that produce dinitrogen gas (Table 3). 

It seems, theoretically, that *Nostoc* spp. have an evolutionary adaptation to convert nitrous oxide in the ambient environment into dinitrogen gas, which can be utilized in subsequent nitrogen fixation, which is an extremely clever biological process. The same enzyme (a putative nitrous oxide reductase) was found to be present in the proteome of the cyanobiont of *Azolla* spp. at 31% identity and 55% coverage (Table 3), showcasing the value of growing *Azolla* in rice fields, where urea is used abundantly as a chemical fertilizer, to negate nitrous oxide emissions.

On the other hand, nitric oxide synthases, which synthesize NO, were found in two filamentous subsections, and are available in plant cyanobionts such as *Nostoc cycadae*. However, nitric oxide synthases are absent in the *Azolla* cyanobiont’s genome/proteome (Table 3). Nevertheless, it is interesting that there is an ad hoc nature to the presence of the nitric oxide synthases, being found in only a few species of *Nostoc* and *Anabaena*, which are highly abundant species of the order Nostocales. Interestingly, the *Nostoc* sp. HK-1 (which establishes a symbiosis with *Cycas* plants), which is a drought-tolerant species of *Nostoc* that has been suggested to be a contender that can survive in extraterrestrial environments, such as on Mars. However, in the absence of lab-based evidence, the possibility of the role of NO in cellular signaling and in the dialogue between plants and microbes cannot be eliminated.

The accumulation of NO, which takes place in *Nostoc* species, appears to be a signaling event between plants and cyanobacteria. Still, there is another enzymatic contender for the production of NO, namely, the family of nitrite reductases. Table 3 presents a summary of the representation of subsections IV and V of cyanobacteria and their complement of nitrogen-based enzymes, in particular, denitrifying proteins. Of note, *N. azollae* also had a low homology match (22% identity and 30% coverage) to nitrite reductases, which requires experimental data to convincingly demonstrate that it is an authentic nitrite reductase (Figure S1).

It is noteworthy that, in the past, scientists questioned whether NO is more important for plants or plant pathogens, since it is exuded by the latter [22]. In a similar context, the presence of NO, but not N_2_O, is a measure of the use of NO in *Nostoc* biology. It is known that microorganisms that are capable of parasitism utilize NO in highly efficient, constitutive, and inducible ways [22]. Therefore, the presence of NO could analogously be a windfall for the *Nostoc* cyanobacteria, especially those capable of symbiotic unions. Thus, similar to being a virulence factor in plant pathogens [22], NO could well be a symbiotic factor in cyanobacteria capable of symbioses. Current knowledge related to this is strictly limited in showcasing all the biological phenomena associated with NO [22].

### 2.3. Cyanobacterial Signaling with Host: New In Silico-Based Evidence on Nod Factors

The key finding of this study is the gap in the denitrification process in *Nostoc* species (Table 3). Still, the presence of nitrite reductases in some *Nostoc* species and nitric oxide synthases in others presents a picture of accumulating NO levels that are not utilized downstream due to the absence of nitric oxide reductases, which prevents the production of nitrous oxide by any *Nostoc* species (Table 3).

Furthermore, I hypothesize that the presence and accumulation of nitric oxide may have a role to play in the establishment of symbioses with host plants. Fascinatingly, *(Ns)* H-NOX is a hemeprotein found in symbiotic cyanobacteria that is markedly similar to the β subunit of soluble guanylyl cyclases (sGC), which indicates a NO-sensing function in cyanobacteria that may use plant-derived NO for various processes [23]. However, it does not explain the de novo synthesis of NO in such cyanobacteria using nitric oxide synthases and nitrite reductases, although the emanation of NO by cyanobacterium can be a mechanism for drawing the root closer toward the cyanobacterium, aided by the elongation of the root.

Considering the similarities between the genera *Frankia* and *Nostoc*, in that both form symbiotic unions with land plants while producing an organ such as a root nodule and possessing the ability to differentiate into a distinct cell/vesicle to fix nitrogen, I checked for the presence of nodulation protein NodA, nodulation protein H, and NodB polysaccharide deacetylase from “*Candidatus* Frankia californiensis” [24] as the search query using the PSI-BLAST search tool. Table 4 clearly shows the presence of NodB polysaccharide deacetylases in cyanobacterial genomes, including that of *Nostoc* spp., which draws a link between two filamentous/mycelial types in two divergent plant systems. What is most interesting is that the NodB polysaccharide deacetylase family is strongly conserved in several cyanobacterial species, especially those of the order Nostocales. 

**Figure 3:**
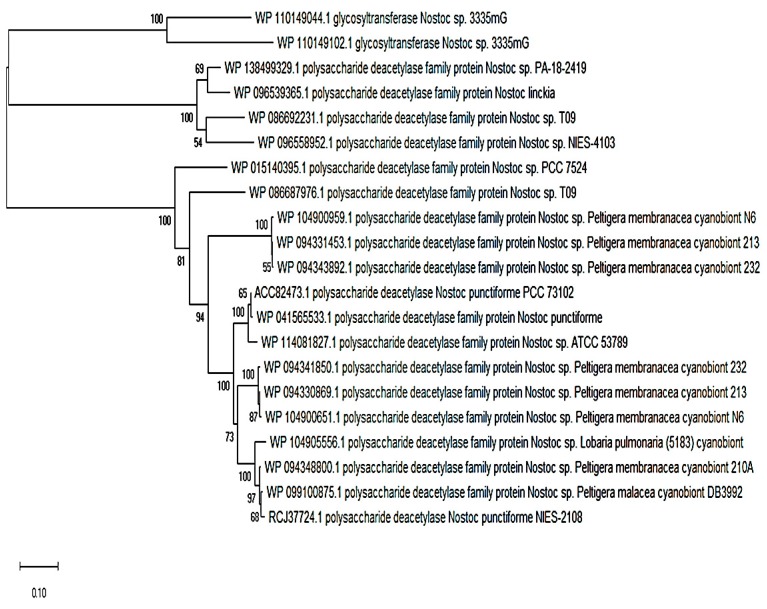
The downloaded amino acid sequences of NodB nodulation proteins that were aligned with the ClustalW algorithm using MEGA version X and the phylogenetic reconstruction performed using the neighborhood joining method with support from 500 bootstrap replications. NodB nodulation proteins are found in symbiotic cyanobacteria (namely, *Nostoc*), but are also available in noncyanobionts

I also further searched the genus *Nostoc* with a NodB polysaccharide deacetylase protein from a rhizobial strain (*Rhizobium leucaenae* USDA 9039) using the PSI-BLAST search tool. I found a myriad of *Nostoc* species that possess homologues of >30% identity and 80% or over sequence coverage. The first 25 hits on the PSI-BLAST search were mostly symbiotically-inclined cyanobionts representing hosts such as lichens, lower plants, gymnosperms, and angiosperms (Figure 3). However, there were NodB polysaccharide deacetylases found in noncyanobionts. 

It is worth considering what made the NodB family of polysaccharide deacetylases important for communication by cyanobacteria, as revealed by their widespread conservation (Figure 3). It seems that when it comes to partnerships such as *Rhizobia*—legumes, actinorhizal-plants—*Frankia*, and host—cyanobacterium symbiotic systems, there is a role to be played by NodB polysaccharide deacetylases, which is clearly shown by the level of sequence conservation in cyanobionts. This family of proteins (carbohydrate esterase family 4 (CE4)) may perform its function by cleaving an *N*-acetyl moiety from the nonreducing end of the chitin oligosaccharide (CO) molecule [25].

Surprisingly, even NodC nodulation proteins (glycosyltransferases) were present in PSI-BLAST queries of a NodC protein (*R. leucaenae* USDA 9039), with the best match being 55% coverage/35% identity (Figure S2). NodC proteins are N-acetylglucosaminyltransferases forming chitin oligosaccharide molecules. Therefore, two sequential steps of the rhizobial nod factor design pathway (NodCand NodB nodulation proteins) are putatively conserved in many *Nostoc* species. 

Surprisingly, the NodB polysaccharide deacetylase protein is found in the cyanobiont of *Azolla* spp. at 90% query cover and 35% sequence identity to the *Rhizobium* query sequence, while the NodC acetylglucosaminyltransferase is found at 34.7% coverage but only 22% identity when searched with the respective proteins of *R. leucaenae* USDA 9039 (Table 5). Whether or not the activities of synthesis and deacetylation of the chitin oligosaccharide molecules take place in *Azolla* spp. is dependent on showing experimentally that they have a function in the symbiosis. This changes our perception of the subject; that is, *N. azollae,* contrary to the notion that it is incapable of horizontal transmission, is perhaps capable of infection, or once had such a capability. 

Upon searching the *Azolla* plant proteome for the presence of the LysM receptor-like kinase (Nod factor perception protein), which mediates Nod factor recognition, I found that they were absent in the genus *Azolla*. Perhaps contrary to the belief that it is the cyanobiont that lost its capacity to infect, I hypothesize here that the *Azolla* plant does not possess LysM receptor-like kinases for Nod factor perception. I suggest that the Nod factors in *N. azollae* are involved in a different, unknown context to mere recognition and infection, which is also supported by the presence of NodB nodulation proteins in noncyanobionts incapable of symbioses.

**Figure 4:**
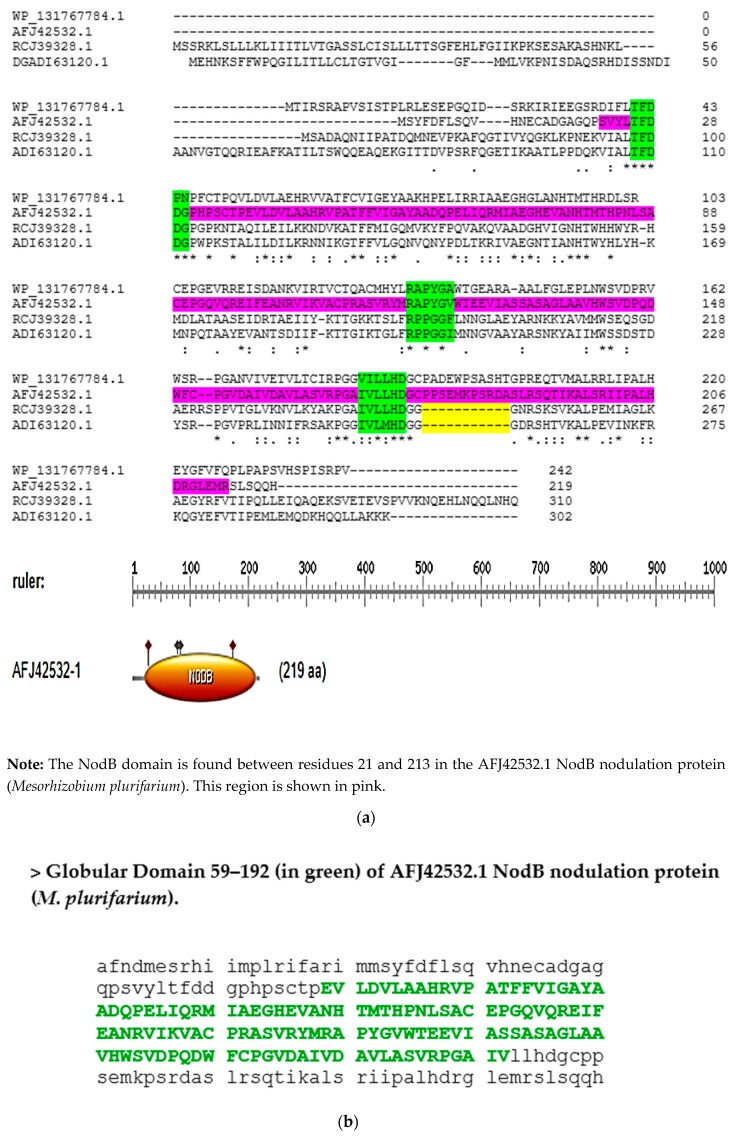

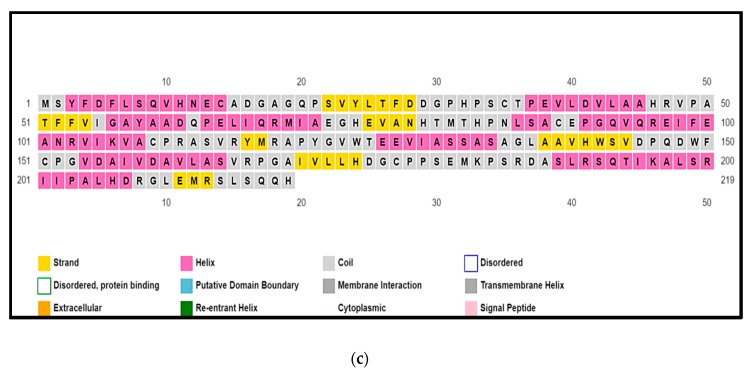
(**a**): ClustalW sequence alignment of four NodB deacetylase proteins: [>WP_131767784.1 chitooligosaccharide deacetylase NodB [*Frankia* symbiont of *Datisca glomerata*]; [>AFJ42532.1 NodB nodulation protein [*M. plurifarium*]; [>RCJ39328.1 polysaccharide deacetylase [*Nostoc punctiforme NIES-2108*]; [>ADI63120.1 polysaccharide deacetylase [*Nostoc azollae* 0708]. **Note:** Shown in green are the 3/5 motifs that form key sites for enzymatic activity. Yellow indicates the interruptions (deletions) in the NodB domain in sequences from cyanobacteria. (**b**) Prediction of the Globular Domain of sequence AFJ42532.1 (NodB nodulation protein from *M. plurifarium*) using http://globplot.embl.de/. The amino acids in green indicate the globular domain, which contains the majority of the catalytic site. (**c**) Output of the secondary structure prediction of sequence AFJ42532.1 (NodB nodulation protein from *M. plurifarium*) using the service PSIPRED 4.0, showcasing helixes, beta strands, and coils.

Using ClustalW, I aligned four sequences of putative NodB polysaccharide deacetylases from an actinorhizal symbiont, a species of *Rhizobium*, and homologues from *N. azollae* and *Nostoc punctiforme* to examine the sequences in detail (Figure 4). The NodB domain was found mostly within the globular fold of the NodB proteins (Figure 4b), which aligned well with its role as the catalytically-competent area of the protein. However, the NodB domain was interrupted (a deleted region) in both cyanobacterial sequences after the fifth conserved motif/patch (Figure 4a), which highlighted the fact that the substrate of cyanobacterial NodB proteins may perhaps be different than the bacterial counterparts in their substrate profiles, or such cyanobacterial proteins are inactivated or pseudogenized at the genome level. The deleted region (Figure 4a) corresponded to a coil region (Figure 4c) when the secondary structural motifs were predicted of the AFJ42532.1 NodB protein (*Mesorhizobium plurifarium*).

### 2.4. Methane Emissions

Cyanobacteria have been shown to produce methane from aquatic and terrestrial ecosystems [26]. It is a stark reality that biogenic methane production by cyanobacteria affects the recent and future methane budgets, and is also part of a protracted time frame (3.5 billion years) since their evolutionary beginnings, where they would have furnished methane to the ambient environment [26]. It is hypothesized that the increased rates of hydrogen production in diazotrophs, such as the cyanobiont *N. azollae,* promote the production of methane through the transfer of hydrogen gas into hydrogenotrophic methanogenic bacteria in the ambient environment [26]. Still, the cultivation of *Azolla* spp. in methane-emanating rice paddies, which are hot spots for methanogenic phenomena, has been shown to alleviate methane emissions in dual cropping systems [27]. The supposed explanation of this observation is that the dissolved oxygen in irrigation systems and the soil redox potential become favorable under *Azolla* cultivation [27].

**Figure 5:**
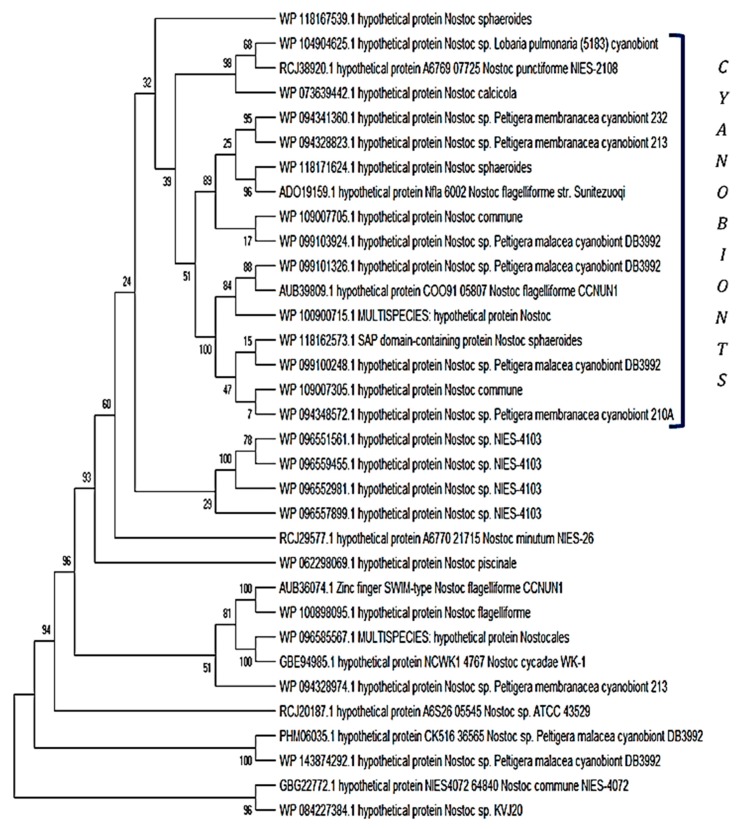
The downloaded amino acid sequences of the methane production catalyzing proteins that were aligned with the ClustalW algorithm using MEGA version X and the phylogenetic reconstruction performed using the maximum parsimony method with support from 500 bootstrap replications. There was no assignment of outgroups. The majority cyanobionts cluster (mixed with noncyanobionts) is shown in a bracket.

I searched cyanobacteria for the presence of methanogenic reactions using the hypothetical protein CEN44_02070 (*Fischerella muscicola* CCMEE 5323), which can synthesize methane from methyl-coenzyme M and coenzyme B through a methyl-coenzyme M reductase (MCR) gamma subunit domain, which is abundant in methanogenic organisms, including cyanobacteria. Again, no homologues of the abovementioned protein were found in the proteome of the *Azolla* cyanobiont (Table 3). I then constructed a phylogenetic tree with some of the best aligned sequences to the hypothetical protein CEN44_02070 (*F. muscicola* CCMEE 5323), where I found an enrichment/abundance of sequences from cyanobionts, from several sources, compared with those of free-living cyanobacteria. In particular, there was a strong representation of the *Nostoc* partners of *Peltigera* lichens in the phylogenetic reconstruction (Figure 5).

In addition, it was recently shown that the wild-type iron–iron (Fe-only) nitrogenase from the bacterium *Rhodopseudomonas palustris* can produce hydrogen, ammonia, and methane in a single enzymatic reaction [28]. Therefore, I also checked this using PSI-BLAST, considering the unlikely possibility of *N. azollae* possessing this rare enzyme (iron–iron nitrogenase), but I failed to identify a candidate enzyme in the database.

### 2.5. Halocarbon Emissions

I searched for vanadium-dependent haloperoxidases that can synthesize halocarbons as by-products from the oxidation of halides, which can damage the ozone layer and contribute to global warming [29]. It is interesting that almost all cyanobionts of plants and lichens have a vanadium nitrogenase as a component of their two-prong nitrogen fixation systems, which makes the partitioning of vanadium to the nitrogenase and haloperoxidases a critical task, when it is present [30,31].

Chloroperoxidases and bromoperoxidases have highly different protein sequences, but are conserved in their catalytic mechanisms, proving that enzyme evolution takes place by modifying the substrate binding site but keeping the coordination chemistry of the active site [32]. I searched the vanadium chloroperoxidase and bromoperoxidase from *Nostoc* sp. PCC 7120 and *Nostoc commune* NIES-4072, respectively, against the *N. azollae* proteome. The former (vanadium chloroperoxidase) yielded the best match at 84% coverage and 48% sequence identity (Table 3), while the latter was not found, showing that vanadium chloroperoxidases may be a potential source of greenhouse gases in *Azolla* spp.

## 3. Conclusions

This bioinformatics study demonstrated the paucity of greenhouse gas emissions by the *Azolla*—cyanobiont symbiosis, namely, nitrous oxide and methane, with the exception of some forms of halocarbons and carbon dioxide, which are emanated by *N. azollae*. It was also shown that the released carbon dioxide can be quenched by the fronds of *Azolla* spp. at the same efficiency and range as other aquatic floating plants and C_3_ counterparts. It was further demonstrated, based on computational biology, that there is nitrous oxide quenching and transformation into dinitrogen gas by the *Azolla* cyanobiont, offering a near-ideal biological system for greenhouse gas mitigation for rice cultivation with extensive urea usage. Cyanobacteria also possess the genomic ability to produce Nod factors, which are, surprisingly, found in *N. azollae* as well.

## 4. The Future

During the *Azolla* event 49 million years ago, *Azolla* blooms in the Arctic Ocean were able to trap carbon dioxide and become sediments on the ocean floor, which changed the earth from a greenhouse into an icehouse [33]. This one event by itself demonstrates the importance of *Azolla* spp. in a future world impacted by climate change, where they could play a crucial role in reversing greenhouse effects and the resulting threat of climate change, which has uprooted lives in island nations such as Sri Lanka. Such places have already witnessed changing weather patterns and the degradation of the overall quality of life. In that stark horizon, there lies a role for the genus *Azolla* to be a frontrunner to combat climate change.

## 5. Materials and Methods

### 5.1. Sequence Queries

Proteins were identified from past literature and searched using the NCBI protein database (https://www.ncbi.nlm.nih.gov/protein) using word-based queries. The most crucial word-based protein searches are provided in Table 2.

I assessed the top ~100 sequences derived from PSI-BLAST searches and downloaded nonredundant sequences for phylogenetic tree construction. Phylogenetic trees played two roles in this study, i.e., providing a visual/nominal identity, and showing the phylogeny of cyanobionts against noncyanobionts. I attempted, when possible, to employ a balance between free-living cyanobacteria and cyanobionts, while eliminating redundant sequences.

The PSI-BLAST query focused on the umbrella taxon “cyanobacteria” using the FASTA sequence of the sequence query to run the search tool (provided in Table 2). Furthermore, for specific queries such as nitric oxide reductases, I employed a “*Nostocales*”-only PSI-BLAST search, which failed to yield any *Nostoc* or *Anabaena* species, even at a low sequence homology level, demonstrating their absence in such genera. This search, in particular, was conducted to ensure that both the genera *Nostoc* and *Anabaena* were without nitric oxide reductases, a key finding of this study. Similarly, more specific searches were also conducted at the genus and species levels according to the impending requirements.

The threshold/cut-off of 30% sequence identity was used as an indicator of members of a single family of proteins according to the Structural Classification of Proteins (SCOP) rules [34]. The 30% coverage was more of an arbitrary value, considering the spans of sequence conservation.

### 5.2. Phylogenetic Reconstructions

The nonredundant downloaded amino acid sequences (as FASTA files) from each query were first aligned with the ClustalW algorithm using MEGA version X (default parameters) [35], which were converted to the MEGA sequence format, and phylogenetic reconstruction was performed using the neighborhood joining/maximum parsimony methods (again in MEGA version X) with support from 500 bootstrap replications. There was no assignment of outgroups, since the phylogenetic trees were used here to showcase the topmost hits (the IDs) and, to a lesser extent, to show phylogeny between cyanobiont sequence clusters.

### 5.3. Secondary Structure Prediction

The secondary structure prediction service PSIPRED 4.0 (http://bioinf.cs.ucl.ac.uk/psipred/) was used to showcase the helixes, beta strands, and coils [36].

### 5.4. Globular Domain Prediction

Globular domains were predicted using the bioinformatics server at http://globplot.embl.de/ [37].

## Figures and Tables

**Table 1:** The spacing of heterocysts in cyanobacteria of different pedigrees. *N. punctiforme* is a horizontally transferred cyanobiont, *Anabaena variabilis* possesses a two-prong nitrogenase system (inclusive of a vanadium nitrogenase), and *Anabaena cylindrica* PCC 7122 is a free-living cyanobacterium. The following were obtained from [18,19] and this study.

Cell Type	Organism	Spacing/Percentage
Heterocyst	*N. azollae*	2–10 cells apart
Heterocyst	*N. punctiforme*	3–10% of the total cells
Heterocyst	*A. cylindrica* PCC 7122	8–15 cells apart
Heterocyst	*A. variabilis*	5–10% of cells

**Table 2:** Query and the PSI-BLAST query employed in this study.

Word Query	PSI-BLAST Query	Type of Cyanobacteria/Bacteria
Nitric Oxide reductase	AFZ55274.1, belonging to *Cyanobacterium aponinum* PCC 10605	Freshwater, Unicellular
Nitrous Oxide reductase	RCJ21339.1, belonging to *Nostoc* sp. ATCC 43529	Subsection IV, Filamentous Cyanobacteria
Methanogenic enzymes	CEN44_02070 (*Fischerella muscicola* CCMEE 5323)	Subsection V, Filamentous Cyanobacteria
NodB protein	CAA67138.1—NodB polysaccharide deacetylase (*Rhizobium leucaenae* USDA 9039)	Bacterial, Nodular
NodC protein	CAA67139.1—N-acetylglucosaminyltransferases (*R. leucaenae* USDA 9039)	Bacterial, Nodular
Chloroperoxidases	CAA04998.1—*Nostoc sp.* PCC 7120	Subsection IV, Filamentous
Bromoperoxidases	GBG19525.1—*Nostoc* *commune* NIES-4072	Subsection IV, Filamentous

**Table 3:** The enzymes that were employed for bioinformatics explorations and their occurrences/distribution. (Yes—sequence identity is >30% and sequence coverage of >30%; No—identity is <30% or sequence coverage <30%).

Protein	Key Enzyme-Derived Product	Subsection V Cyanobacteria	Subsection IV Cyanobacteria	*Azolla* Major Cyanobiont
Nitrite reductase	Nitric Oxide	YES	YES	NO
Nitric Oxide (NO) synthase	Nitric Oxide	NO	YES	NO
Nitric Oxide (NO) reductase	Nitrous Oxide	YES	NO	NO
Nitrous Oxide (N_2_O) reductase	Nitrogen gas	YES	YES	YES
Methanogenic enzyme	Methane	YES	YES	NO
Vanadium dependent Chloroperoxidase	Halocarbons	YES	YES	YES
Vanadium dependent Bromoperoxidase	Halocarbons	YES	YES	NO

**Table 4:** Search results of Nod-factor-producing/modifying proteins in all cyanobacteria and in the genus *Nostoc*, using “*Candidatus* Frankia californiensis” Nod-factor-related sequences as queries.

Protein	Best Match in Cyanobacteria /Coverage/Sequence Identity	Best Match in *Nostoc*/Coverage/Sequence Identity
Nodulation protein A	RsmB/NOP family class I SAM-dependent RNA methyltransferase [*Scytonema tolypothrichoides*]; 41%; 24.69%	transcriptional regulator [*Nostoc* sp. MBR 210]; 40%; 26.25%
Nodulation protein H	type I polyketide synthase [*Calothrix brevissima*]; 22%; 31.75%	glycosyl transferase [*Nostoc* sp. ATCC 53789]; 17%; 34.09%
NodB polysaccharide deacetylases	Polysaccharide Deacetylase family protein [*Fischerella* sp. PCC 9605]; 86%; 35.8%	Deacteylase NodB [*Nostoc* sp. 3335mG]; 81%; 37.44%

**Table 5:** Search results of Nod-factor-producing/modifying proteins in the *Azolla* cyanobiont’s proteome using the relevant proteins from *R. leucaenae* USDA 9039 as the query proteins.

*Azolla* Cyanobiont’s Protein Type	Sequence Identity to the Homolog in *R. leucaenae* USDA 9039	Coverage
NodB polysaccharide deacetylase	35%	90%
NodC acetylglucosaminyltransferase	34.7%	22%

## Supplementary Materials

The following are available online at https://www.mdpi.com/2223-7747/8/12/587/s1, Figure S1: Screenshot of PSI-BLAST result for Nitrite Reductases in N. azollae., Figure S2: Screenshot of PSI-BLAST query result, when a single NodC nodulation protein from *R. leucaenae* USDA 9039 was searched specifically against cyanobacteria.
