# Supplementary material for: An Exploration of Common Greenhouse Gas Emissions by the Cyanobiont of the Azolla–Nostoc Symbiosis and Clues as to Nod Factors in Cyanobacteria
Source: Plants (Basel). 2019 Dec 10;8(12):587. doi: 10.3390/plants8120587 (PMC6963936; doi:10.3390/plants8120587)
Supplement: Supplementary file 1 [file plants-08-00587-s001.pdf]

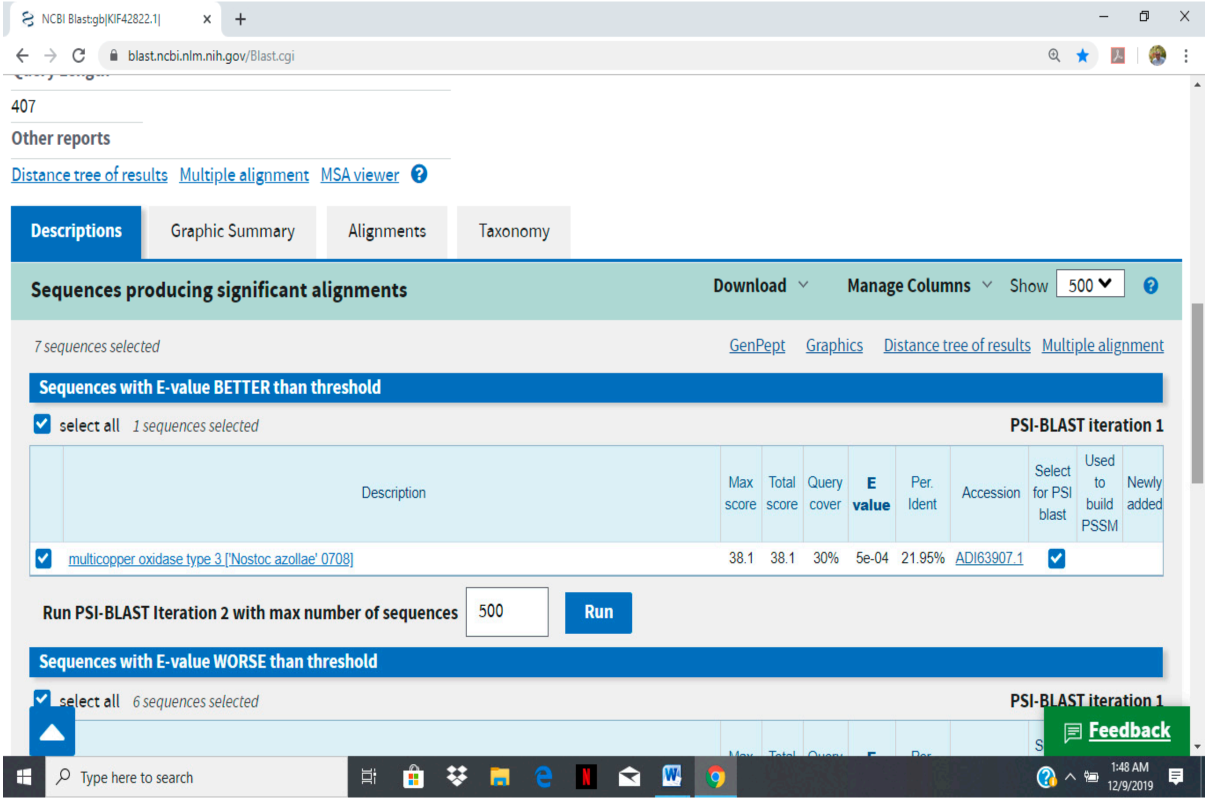

Figure S1. Screenshot of PSI-BLAST result for Nitrite Reductases in *N. azollae*.

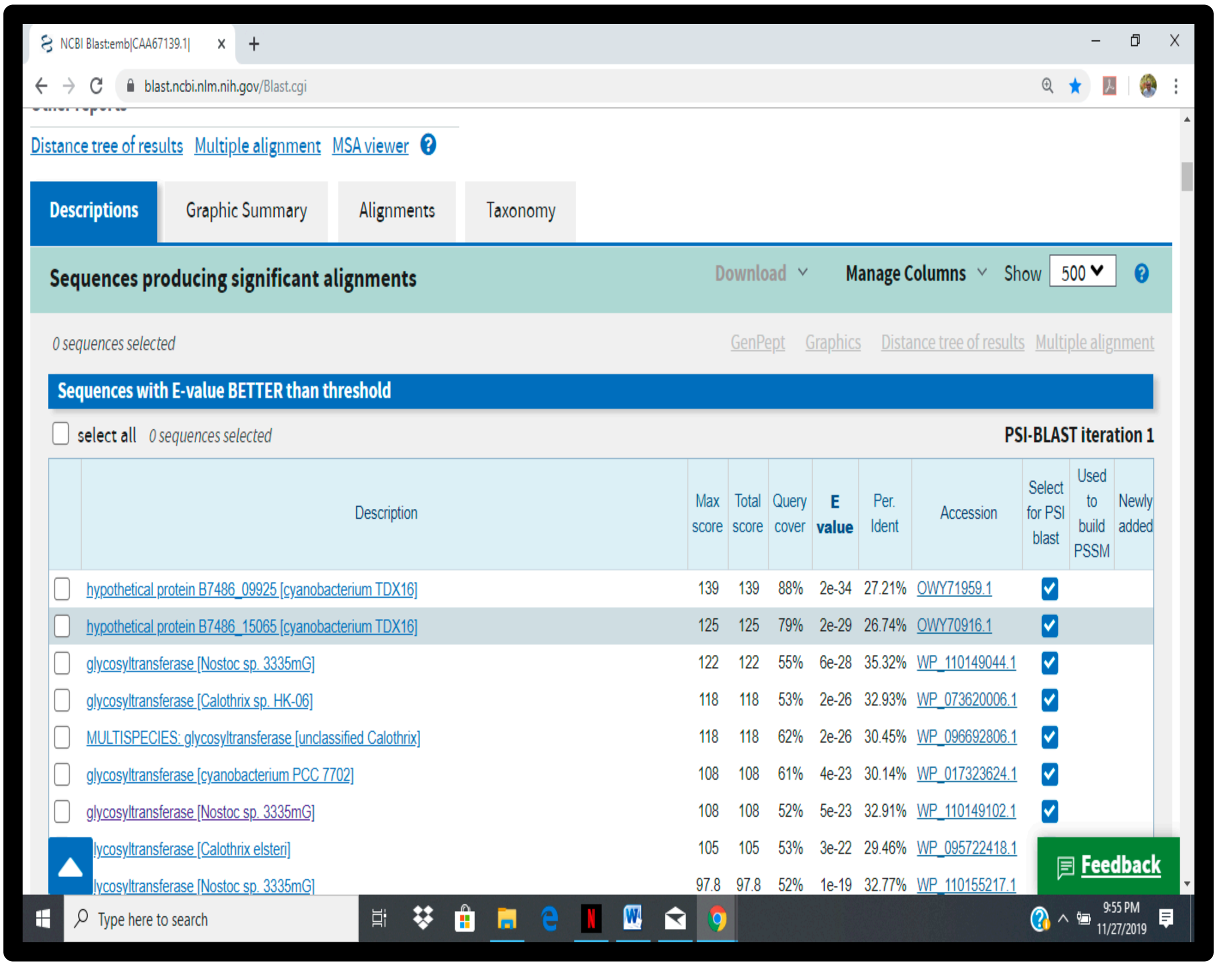

Figure S2. Screenshot of PSI-BLAST query result when NodC nodulation protein from *R. leucaenae* USDA 9039 was searched, specifically on cyanobacteria.
